# The Role of SGLT2 Inhibitor on the Treatment of Diabetic Retinopathy

**DOI:** 10.1155/2020/8867875

**Published:** 2020-11-12

**Authors:** Wenjun Sha, Song Wen, Lin Chen, Bilin Xu, Tao Lei, Ligang Zhou

**Affiliations:** ^1^Department of Endocrinology and Metabolism, Putuo Hospital, Shanghai University of Traditional Chinese Medicine, Shanghai 200062, China; ^2^Department of Endocrinology, Shanghai Pudong Hospital, Fudan University, Shanghai 201399, China

## Abstract

Diabetic retinopathy (DR) is one of the most serious complications of diabetic microangiopathy. DR has an early onset and is not easy to detect. When visual impairment occurs, the optimal period for therapy is often missed. Therefore, the prevention and treatment of DR should start from the early stage of diabetes. Sodium-dependent glucose transporter 2 inhibitor (SGLT2i) is a new antidiabetic drug which is mainly used in clinical practice to control blood glucose of patients with type 2 diabetes prone to develop chronic heart failure. Recent studies have found that SGLT2 is also expressed in the human retina. Now, the prevention and treatment of diabetic retinopathy with SGLT2i while reducing blood sugar has become a new research field. Hence, this article reviewed the recent therapeutic and research progress of SGLT2 in the treatment of diabetic retinopathy.

## 1. Introduction

Diabetes is a group of metabolic diseases characterized by hyperglycemia and microvascular and macrovascular complications caused by insulin secretion defect and/or its biological function disorder [1–3]. According to the International Diabetes Federation (IDF), there were 425 million people with diabetes aged 20-79 worldwide in 2017, and the number will increase to 629 million at the middle of this century [4]. Diabetic retinopathy (DR) is one of the most common and serious microvascular complications in diabetes. Studies demonstrate that the incidence of DR increases with the course of diabetes.

An epidemiological survey in 2011 showed that the incidence of DR is about 25% of 5 years after the diagnosis of diabetes, 60% after 10 years of it, and up to 75%~80% after 15 years of it [5]. At the same time, DR is also the main cause of visual impairment and irreversible blindness in middle-aged and elderly people. According to the data published by the World Health Organization in 2002, DR worldwide caused about 4.8% of the 37 million cases of blindness [6].

## 2. The Classification of Diabetic Retinopathy

The classification of DR development is related to the abnormalities of retinal microvascular system, including increased blood retinal barrier permeability, decreased vascular endothelial cells and pericytes, thickened vascular basement membrane, capillary occlusion, retinal neurons, and glial abnormalities. Therefore, DR is currently divided into nonproliferative diabetic retinopathy (NPDR) and proliferative diabetic retinopathy (PDR).

### 2.1. Nonproliferative Diabetic Retinopathy (NPDR)

Nonproliferative diabetic retinopathy (NPDR) occurs in the early stage of DR. The main pathophysiological basis is high glucose-induced retinal degeneration, including loss of capillary pericytes, thickening of the basement membrane, thinning of the blood vessel layer and destruction of the blood-retinal barrier, changes in the broken line leading to retinal hemorrhage and arteriole tumors, and microvascular abnormalities in the retina and cotton wool spots (fluffy white plaques on the retina caused by local swelling of the retinal nerve fiber layer) [7]. However, in the NPDR stage, patients are usually asymptomatic and have normal vision. When visual impairment occurs, the best period of treatment is often missed [8]. Therefore, it is necessary to prevent the occurrence of DR in the early stage of diabetes.

### 2.2. Proliferative Diabetic Retinopathy (PDR)

Studies have found that the prevalence of PDR is close to 50% after 25 years of diabetes diagnosis, and most patients with type 1 diabetes (T1DM) will develop PDR after about 10 years [9]. At the PDR stage, the new blood vessels in the eye are continuously generated and often accompanied by the oozing and hyperplasia of the eye tissue, which can destroy the normal structure and function of the eye and ultimately lead to the visual impairment of the patient [10]. Compared with NPDR, PDR is more harmful to eyesight and could cause severe vision loss or even complete blindness.

## 3. The Mechanism for Diabetic Retinopathy

**Figure 1:**
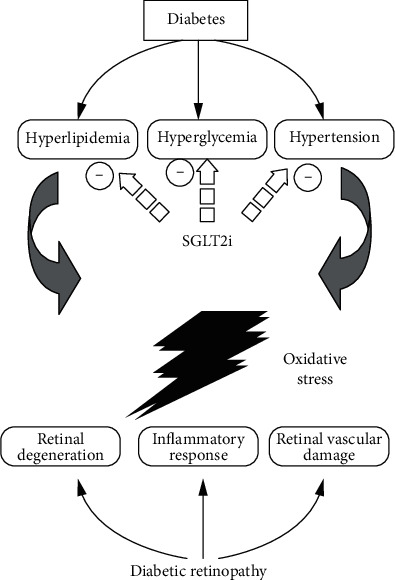
The proposed mechanism of SGLT2 inhibitor (SGLT2i) against diabetic retinopathy, in which SGLT2i improves hyperlipidemia, hyperglycemia, and hypertension.

The pathogenesis of DR is complex, and the mechanism has not yet been fully elucidated. The pathological mechanism hypotheses currently proposed mainly include the theory of chronic inflammation, retinal hemodynamic changes, oxidative stress, gene polymorphism, and neurodegenerative changes (Figure 1).

### 3.1. Inflammation

Chronic hyperglycemia leads to oxidative damage of retinal capillary wall and vascular occlusion by activating various metabolic pathways. In the early stage of hyperglycemia, transcription factors are activated to elevate proinflammatory factors, triggering the activation of microglia cells and resulting in low-grade inflammation on the retina [11]. Secondly, the increase of icAM-1 expression in retinal endothelial cells and the accumulation of white blood cells around the retinal capillary walls will cause the retina to produce low-grade inflammation through the release of cytokines, chemokines, proinflammatory factors, and proangiogenic growth factors [12].

This low-grade inflammation destroys the tight junctions between endothelial cells by pericyte injury [13], increasing the permeability of blood vessels, exuding serum and weakening the vascular wall barrier, destroying the retinal blood barrier (BRB), and causing edema in the retina. Because of edema, the extracellular hydrostatic pressure increases, causing the capillary network to collapse and increase occlusion, leading to increased retinal ischemia, glycosylation, thickening of the endothelial basement membrane, development of vascular malformations, and formation of microaneurysms [11].

### 3.2. Oxidative Stress and Free Radicals

There are a large number of polyunsaturated fatty acids on the retina, which makes it particularly sensitive to oxidative stress [14]. In the process of glucose oxidation, nonenzymatic saccharification of proteins occurs and reactive oxygen species (ROS) is produced. Hyperglycemia leads to an increase in the synthesis of ROS in the retina, which acts as a coinitiating factor to activate four classical molecular pathways [15], namely, abnormal polyol pathway, inositol metabolism pathway, accumulation of protein nonenzymatic glycosylation end products [16], and protein kinase C (PKC) activates [17] and acts on the angiotensin-converting enzyme system [18]. The activation of each pathway further promotes the generation of ROS with positive feedback, which enhances oxidation and damages the blood-retinal barrier structure, which ultimately leads to the occurrence of DR.

## 4. The Treatment of New Diabetic Drug SGLT2 Inhibitor on the DR Treatment

In 1835, Petersen extracted Phlorizin, the nonselective sodium-dependent glucose transporters (SGLT) inhibitor from apple roots [19]. In further animal models, it was found to increase urine glucose excretion and reduce hyperglycemia. However, Phlorizin is prone to cause adverse reactions of the digestive tract and has low utilization efficiency, so it is not used in the treatment of diabetes [20]. Modern studies have found that SGLT2 belongs to the superfamily of sodium-glucose cotransporters. It is a low-affinity, high-efficiency transporter. It is not only distributed in the proximal convoluted tubules S1 and S2 in the kidney but also in the lens and the retina [21]. SGLT2 provides protection for the subtle nutrient metabolism of the eye [22]. Based on this, sodium-glucose transport synergistic protein 2 inhibitor (SGLT2i) is independent of hypoglycemic effect and also has DR protective effect.

### 4.1. Improvement of Factors of Diabetic Pathogenesis

There are many risk factors affecting the occurrence of DR, including poor blood sugar control, hypertension, and hyperlipidemia. Improving these influence factors can effectively delay the progress of DR [23].

#### 4.1.1. Control of Hyperglycemia

It is known that the microvascular complications of type 2 diabetes (T2DM) are related to hyperglycemia. UKPDS has shown that a 1% reduction in HbA1c can reduce the risk of microvascular complications by 37% [24]. For every 1 mmol/L decrease in blood glucose level, the risk of DR will decrease by 21% [25]. In the “Diagnostic Standards for Diabetes Medicine” issued by the American Diabetes Association (ADA) in 2020, the new hypoglycemic drug SGLT2i is recommended as a first-line drug in the patients with chronic heart failure. In the first week of treatment, it can reduce fasting blood glucose by about 1.5 mmol/L and effectively reduce HbA1c by about 1.5%, which is equivalent to the efficacy of metformin 2000 mg per day. Due to its multiple benefits in addition to hypoglycemic effect, SGLT2i could also be administrated in appropriate diabetic patients [26].

#### 4.1.2. Control of Hypertension

In T2DM patients with hypertension, strict control of blood pressure can delay the progression of DR and the deterioration of visual acuity [27]. Studies have confirmed that for every 10 mmHg drop in systolic blood pressure (SBP), the risk of diabetic microvascular complications can be reduced by 13%, and strict blood pressure control can reduce the risk of blindness in DR by 47% [28]. Elevated 24-hour SBP is independently associated with higher wall-to-lumen ratio (WLR) in retinal arterioles [29]. A clinical study involving 311 centers in 16 countries has confirmed that SGLT2i has a significant antihypertensive effect, which can reduce SBP by 11.9 mmHg on average. SGLT2i combined with other common hypertension drugs can achieve more satisfactory antihypertensive effect [30]. At present, some scholars believe that the antihypertensive mechanism of SGLT2i should be related to the osmotic diuresis caused by glucose excretion [31]. Some other scholars also believe that its antihypertensive effect is related to the inhibitory effect on the renin-angiotensin-aldosterone system when promoting urine excretion. Dapagliflozin improves muscle insulin sensitivity but enhances endogenous glucose production [32, 33]. Clinical studies found that BP parameters were lower than baseline 6 weeks after Dagliazine treatment. Therefore, Dagliazine may prevent retinal wall remodeling by lowering BP [34].

#### 4.1.3. Control of Hyperlipidemia

Dyslipidemia also promotes the development of DR. The hard exudation of DR is the exudate of lipid and protein that occurs in the plexiform layer of the retina. It has been found that the severity of hard exudation of DR is positively correlated with triglyceride (TG), low-density lipoprotein (LDL), and total cholesterol (TC) levels and negatively correlated with high-density lipoprotein (HDL) levels [35]. Blood lipid control can improve the function of retinal vascular endothelial cells, reduce inflammation and leakage of fundus microvessels, and thus delay the development of DR [36]. The effect of SGLT2i on blood lipids is multifold. It can be observed that total cholesterol increase/no change, triacylglycerol decrease/no change, and LDL-C and high-density lipoprotein cholesterol (HDL-C) increase by 5%~10%, but LDL-C/HDL-C ratio is not affected [37]. In addition, small and dense LDL-C was reduced by 20%~30% after SGLT2i treatment [38].

### 4.2. Protection of Blood-Retinal Barrier

DR is characterized by the destruction of the blood-retinal barrier (BRB) and leakage of retinal vascular. Normal retina has two layers, an inner and outer BRB. There are two types of BRBs in the normal retina. The inner barrier is composed of zonula occludens between retinal capillary endothelial cells and pericytes; and the outer barrier is composed of retinal pigment epithelium (RPE) layer and the small closed zone between them. Hyperglycemia, ischemia, hypoxia, oxidative stress, inflammation, and inflammatory factors are the main predisposing factors for the destruction of BRB caused by diabetes [39]. The destruction of the inner BRB caused by DR is the main cause of vascular leakage.

SGLT2 acts as a glucose sensor in the retinal microvasculature. Excessive entry of Na^+^-dependent glucose can cause intracellular swelling of pericytes [40] and lead to loss of contractile function, death of pericytes, and overperfusion of the retina. The overexpression of the extracellular matrix (such as fibronectin, collagen IV, and laminin) is associated with the thickening of the basement membrane, subsequent microvascular occlusion, and insufficient retinal perfusion [41]. This change in retinal hemodynamics can lead to multiple downstream triggers of DR. SGLT2 inhibitors have been shown to reduce pericyte swelling and extracellular matrix overexpression [42]. Early suppression of hemodynamic changes in DR is of great significance for the development of effective DR treatments [43].

Takakura and others have shown the effect of iplegliflozin on the diabetic retina of spontaneously diabetic Torii obese rats. In this study, diabetic rats treated with iplegliflozin showed a decrease in the oscillating potential on the electroretinogram, the outer nuclear layer of the neural retina was irregular, and iplegliflozin inhibited the progression of lens cataract formation [44].

### 4.3. Protection of the Fundus Capillaries

Arteriole remodeling is a factor leading to the progression of diabetic retinopathy [45]. In PDR, the basement membrane of retinal capillaries thickens, pericytes gradually disappear, microangiomas form, endothelial cells proliferate, luminal occlusion, and finally new blood vessels form. Many increases in WLR and cross-sectional area are characteristic of vascular hypertrophy [46]. Multiple clinical and animal studies have shown that the occurrence and development of DR are related to the increase of WLR and cross-sectional area of arteriolar walls. Grunwald et al. found that in patients with type 1 diabetes and background retinopathy with poor blood glucose control, retinal total volume blood flow was 23% higher than normal [47]. Patel et al. found that compared with patients without diabetes, diabetic patients with signs of diabetic retinopathy had higher blood flow and larger blood vessel diameter [48]. This increased blood flow may cause vascular damage by increasing shear stress, leading to endothelial dysfunction, basement membrane disruption, and extracellular matrix remodeling [49].

A prospective, single-center, placebo-controlled, double-blind, randomised crossover phase IIIb study evaluated the effects of dapagliflozin on the retinal microvasculature, showing that compared with the placebo group, dagrazine treatment group did not increase the retinal wall-cavity ratio and reduced retinal capillary flow, suggesting that dagrazine could prevent vascular hyperemia [50]. In addition, the treatment seemed to prevent structural changes in retinal arterioles. This may be due in part to the drug's ability to reduce glucose resulting in reduced blood flow, reduced subcutaneous fibrin deposition, altered smooth muscle cells, and prevented arteriolar wall thickening [51].

### 4.4. Protection of the Optic Nerve

The optic nerve is a special somatosensory nerve, which is very sensitive to ischemia and hypoxia. The optic nerve is made up of ganglion cell axons. The surface of its nerve fibers has only myelin sheath and no nerve membrane. Once it undergoes apoptosis, it is difficult to regenerate. The progressive apoptosis of optic nerve cells is the main cause of irreversible damage to visual function. There are roughly three most important mechanisms for neurodegeneration in patients with DR: accumulation of extracellular glutamate, oxidative stress, and reduction of neuroprotective factors synthesized by the retina [52].

Recent findings indicate that there is a correlation between SGLT2 expression and the sympathetic nervous system. Hyperfunction of the sympathetic nervous system (SNS) is a feature of obesity and T2DM [53]. We showed for the first time that norepinephrine, the main neurotransmitter of SNS, upregulates the expression of SGLT2 protein in human proximal renal tubular cells [54]. In addition, high-fat diet-fed mice treated with dapagliflozin showed decreased tissue norepinephrine content and tyrosine hydroxylase expression in both heart and kidney tissues, indicating SGLT2 inhibitors in our animal model. It has significant sympathetic inhibition [55]. So far, limited studies have studied the autonomic nervous system associated with DR, and these studies have shown that DR is associated with early autonomic dysfunction in patients with T1D and T2D [56]. The balance between sympathetic and parasympathetic effects determines the normal function of the autonomic nervous system. We have generated new data highlighting that in a mouse model of neuropathic hypertension with substantially activated SNS, nerve damage in the outer layer of the retina is obvious, which is DR. Therefore, SGLT2i may also reduce harmful retinal changes that may be supported by SNS overactivation.

To sum up, SGLT2i has been widely used in the clinical treatment of diabetes. However, the data on their efficacy in diabetic complications, especially in the treatment of DR, were mainly derived from animal experiments. These animal experimental data and conclusions allow us to see the great clinical application potential of SGLT2i in the treatment of DR. It is believed that with more clinical studies on SGLT2i, clinical data on the efficacy of DR will be more abundant.
